# Evidence from an Applied Health Research Question (AHRQ): Health care utilization of publicly funded rehab services for patients post COVID-19 diagnosis.

**DOI:** 10.23889/ijpds.v7i3.2083

**Published:** 2022-08-25

**Authors:** Haley Golding, Katie Churchill, Charissa Levy, Diana An, Ruth Hall, Lesley Plumptre

**Affiliations:** 1 ICES; 2 Rehabilitative Care Alliance

## Objectives

The Rehabilitative Care Alliance issued an Applied Health Research Question request to collect information regarding healthcare and rehabilitation use among COVID-19 positive individuals. The objective of this project is to determine the association between length of stay (LOS) in acute care and the number of rehabilitation services used post COVID-19 diagnosis.

## Approach

Hospital and rehabilitation service use was identified among individuals diagnosed with COVID-19, using administrative health data. Admission into acute care within 30 days post COVID-19 diagnosis was recorded. Use of inpatient, physiatry and home-care rehabilitative services were collected until March 31st 2021. Outpatient rehabilitation reporting is not mandatory and was not included. Marginalization was evaluated using the Ontario Marginalization Index factor scores. The association between LOS in acute care and number of rehabilitation categories used was assessed using a negative binomial model, stratified by with or without a stay in the ICU and controlling for age, sex, comorbidities and long-term care residence.

## Results

Of 181,139 individuals diagnosed with COVID-19 prior to December 31st 2020, 5% were hospitalized. Of those hospitalized 2.3% then entered rehabilitation compared to 0.06% who were not hospitalized post COVID-19 infection. Rehabilitation users had higher residential instability (mean=0.45 vs -0.01 in the overall cohort), dependency (mean=-0.02 vs -0.27) and material deprivation (mean=0.37 vs 0.19) but similar ethnic diversity (mean=0.87 vs 0.90) compared to the full cohort. LOS in acute care was associated with a 3.3% increased risk of using additional rehabilitation services for individuals without a stay in the ICU (RR 1.033, 95% CI: 1.011 to 1.055; p=0.0036), and a 3.7% increased risk for individuals with a stay in the ICU (RR 1.037, 95% CI: 1.025 to 1.048; p<.0001).

## Conclusion

Post COVID-19 diagnosis, a larger proportion of rehabilitation service users were hospitalized compared to all COVID-19+ individuals. Additionally, LOS in acute care was associated with the use of more rehabilitation care categories following a COVID-19 diagnosis, and the association was stronger for more severe cases requiring an ICU stay.

